# Interim Estimates of 2024–2025 COVID-19 Vaccine Effectiveness Among Adults Aged ≥18 Years — VISION and IVY Networks, September 2024–January 2025

**DOI:** 10.15585/mmwr.mm7406a1

**Published:** 2025-02-27

**Authors:** Ruth Link-Gelles, Sean Chickery, Alexander Webber, Toan C. Ong, Elizabeth A.K. Rowley, Malini B. DeSilva, Kristin Dascomb, Stephanie A. Irving, Nicola P. Klein, Shaun J. Grannis, Michelle A. Barron, Sarah E. Reese, Charlene McEvoy, Tamara Sheffield, Allison L. Naleway, Ousseny Zerbo, Colin Rogerson, Wesley H. Self, Yuwei Zhu, Adam S. Lauring, Emily T. Martin, Ithan D. Peltan, Adit A. Ginde, Nicholas M. Mohr, Kevin W. Gibbs, David N. Hager, Matthew E. Prekker, Amira Mohamed, Nicholas Johnson, Jay S. Steingrub, Akram Khan, Jamie R. Felzer, Abhijit Duggal, Jennifer G. Wilson, Nida Qadir, Christopher Mallow, Jennie H. Kwon, Cristie Columbus, Ivana A. Vaughn, Basmah Safdar, Jarrod M. Mosier, Estelle S. Harris, James D. Chappell, Natasha Halasa, Cassandra Johnson, Karthik Natarajan, Nathaniel M. Lewis, Sascha Ellington, Emily L. Reeves, Jennifer DeCuir, Meredith McMorrow, Clinton R. Paden, Amanda B. Payne, Fatimah S. Dawood, Diya Surie, CDC COVID-19 Vaccine Effectiveness Collaborators, CDC COVID-19 Vaccine Effectiveness Collaborators, Joshua Acidera, Laura Aguilar Marquez, Omobosola Akinsete, Harith Ali, Erika Alor, Ike Appleton, Julie Arndorfer, Olivia Arter, Sarah W. Ball, Jaskiran Bansal, Anna Barrow, Leonard Basobas, Adrienne Baughman, Paul W. Blair, Lawrence Block, Bryce Bosworth, Noah Brazer, Genesis Briceno, Daniel Bride, Samuel M. Brown, Sydney Buehrig, Ashley Bychkowski, Sukantha Chandresakaran, Steven Y. Chang, Catia Chavez, Dylan Clark, Sydney A. Cornelison, Jonathan M. Davis, Brian E. Dixon, Thomas J. Duszynski, Inih Essien, Yvette Evans, William F. Fadel, Cathy Fairfield, Courtney Feitsam, Samantha Ferguson, Tammy Fisher, Omai Garner, Sheila Gasparek, Heath Gibbs, Daniela Gonzalez, Alexandra June Gordon, Anirudh Goyal, Carlos G. Grijalva, Sydney Guthrie-Baker, Jacob Hampton, John Hansen, Ebaad Haq, Adrian Hernandez-Frausto, Kinsley Hubel, Mariana Hurutado-Rodriguez, Cameron Hypes, Karen B. Jacobson, Milad Karami Jouzestani, Sindhuja Koneru, Padma Koppolu, Olivia Krol, Lily Lau, Jenna Lumpkin, Karen Lutrick, Cara T. Lwin, Kevin Ma, Kimberly Manchester, Denisse Mariscal, Amanda Martinez, David Mayer, Rylie McBride, David McDonald, Maile McKeown, Sachina Mensah, Nicholas Mohr, Paul Nassar, Caroline O'Neil, Josh Van Otterloo, Elianora Ovchiyan, Bijal Parikh, Jose Pena, Cynthia Perez, Gabriela Perez, Vanessa Pitre, Edvinas Pocius, Jacob Rademacher, Mayur Ramesh, Caitlin Ray, Carolina Rivas, Safa Saeed, Akshay Saluja, Elizabeth Salvagio Campbell, Carleigh Samuels, Maria Santana-Garces, Tanisha Shack, Samantha Simon, Ine Sohn, Vasisht Srinivasan, Hannah Strait, Amy Sullivan, H. Keipp Talbot, Grace Kyin-ye Tam, Shruti Tirumala, Cody Tran, Emily Tribbett, Alyssa Valencia, Ivan Valesquez, Lucy Vogt, Kim Vu, Francesca Yerbic, Arda Yigitkanli, Anne Zepeski

**Affiliations:** ^1^Coronavirus and Other Respiratory Viruses Division, National Center for Immunization and Respiratory Diseases, CDC; ^2^Westat, Rockville, Maryland; ^3^University of Colorado School of Medicine, Aurora, Colorado; ^4^HealthPartners Institute, Minneapolis, Minnesota; ^5^Division of Infectious Diseases and Clinical Epidemiology, Intermountain Health, Salt Lake City, Utah; ^6^Kaiser Permanente Center for Health Research, Portland, Oregon; ^7^Kaiser Permanente Vaccine Study Center, Kaiser Permanente Northern California Division of Research, Oakland, California; ^8^Indiana University School of Medicine, Indianapolis, Indiana; ^9^Regenstrief Institute Center for Biomedical Informatics, Indianapolis, Indiana; ^10^Department of Pediatrics, School of Medicine, Indiana University, Indianapolis, Indiana; ^11^Vanderbilt University Medical Center, Nashville, Tennessee; ^12^University of Michigan, Ann Arbor, Michigan; ^13^Intermountain Medical Center, Murray, Utah; ^14^University of Utah, Salt Lake City, Utah; ^15^University of Colorado School of Medicine, Aurora, Colorado; ^16^University of Iowa, Iowa City, Iowa; ^17^Wake Forest School of Medicine, Winston-Salem, North Carolina; ^18^Johns Hopkins University School of Medicine, Baltimore, Maryland; ^19^Hennepin County Medical Center, Minneapolis, Minnesota; ^20^Montefiore Medical Center, Albert Einstein College of Medicine, Bronx, New York; ^21^University of Washington, Seattle, Washington; ^22^Baystate Medical Center, Springfield, Massachusetts; ^23^Oregon Health and Sciences University, Portland, Oregon; ^24^Emory University, Atlanta, Georgia; ^25^Cleveland Clinic, Cleveland, Ohio; ^26^Stanford University School of Medicine, Stanford, California; ^27^University of California-Los Angeles, Los Angeles, California; ^28^University of Miami, Miami, Florida; ^29^Washington University, St. Louis, Missouri; ^30^Baylor Scott & White Health, Dallas, Texas; ^31^Texas A&M University College of Medicine, Dallas, Texas; ^32^Henry Ford Health, Detroit, Michigan; ^33^Yale University School of Medicine, New Haven, Connecticut; ^34^University of Arizona, Tucson, Arizona; ^35^Department of Biomedical Informatics, Columbia University Irving Medical Center, New York, New York; ^36^New York-Presbyterian Hospital, New York, New York; ^37^Influenza Division, National Center for Immunization and Respiratory Diseases, CDC.; University of Washington/Harborview; University of Colorado; HealthPartners Institute; Johns Hopkins University; University of Colorado; University of Iowa; Intermountain Health; Washington University School of Medicine; Westat, Inc; Henry Ford Health; University of Colorado; Stanford University; Vanderbilt University Medical Center; Vanderbilt University Medical Center; Kaiser Permanente Northern California; University of Utah; Yale School of Medicine; Oregon Health & Science University; Intermountain Health; Stanford University; Baylor Scott & White Health, Baylor University Medical Center, Dallas; Baylor Scott & White Health, Baylor University Medical Center, Dallas; University of California Los Angeles; University of California Los Angeles; University of Colorado School of Medicine; University of Washington/Harborview; Vanderbilt University Medical Center; Westat, Inc.; Regenstrief Institute Center for Biomedical Informatics and Richard M Fairbanks School of Public Health; Regenstrief Institute Center for Biomedical Informatics and Richard M Fairbanks School of Public Health; HealthPartners Institute; University of Colorado; Regenstrief Institute Center for Biomedical Informatics and Richard M Fairbanks School of Public Health; University of Iowa; University of Iowa; Stanford University; Baylor Scott & White Health, Baylor University Medical Center, Dallas; University of California Los Angeles; Baylor Scott & White Health, Baylor University Medical Center, Dallas; University of Iowa; Baylor Scott & White Health, Baylor University Medical Center, Dallas; Stanford University; Yale School of Medicine; Vanderbilt University Medical Center; Stanford University; University of Iowa; Kaiser Permanente Northern California; Oregon Health & Science University; Oregon Health & Science University; Oregon Health & Science University; Baylor Scott & White Health, Baylor University Medical Center, Dallas; University of Arizona; Kaiser Permanente Northern California; Oregon Health & Science University; Henry Ford Health; Kaiser Permanente Center for Health Research; Oregon Health & Science University; Stanford University; Stanford University; University of Arizona; Vanderbilt University Medical Center; CDC; Yale School of Medicine; Baylor Scott & White Health, Baylor University Medical Center, Dallas; University of Colorado; University of Colorado School of Medicine; University of Utah; Washington University School of Medicine; University of Washington/Harborview; Washington University School of Medicine; University of Iowa; University of Iowa; Washington University School of Medicine; Intermountain Health; Washington University School of Medicine; Washington University School of Medicine; Oregon Health & Science University; Stanford University; Baylor Scott & White Health, Baylor University Medical Center, Dallas; Stanford University; Oregon Health & Science University; University of Colorado; Henry Ford Health; CDC and Goldbelt Professional Services LLC; University of Miami; Johns Hopkins University; Washington University School of Medicine; University of Arizona; Washington University School of Medicine; Henry Ford Health; Henry Ford Health; University of Colorado; Vanderbilt University Medical Center; University of Washington/Harborview; Wake Forest University; University of Colorado; Vanderbilt University Medical Center; Stanford University; Henry Ford Health; University of California Los Angeles; Oregon Health & Science University; Washington University School of Medicine; Yale School of Medicine; Washington University School of Medicine; Washington University School of Medicine; Washington University School of Medicine; Yale School of Medicine; University of Iowa

**Summary**

**What is already known about this topic?**

In June 2024, CDC’s Advisory Committee on Immunization Practices (ACIP) recommended 2024–2025 COVID-19 vaccination for all persons aged ≥6 months to provide additional protection against severe COVID-19.

**What is added by this report*?***

Vaccine effectiveness (VE) of 2024–2025 COVID-19 vaccine was 33% against COVID-19–associated emergency department (ED) or urgent care (UC) visits among adults aged ≥18 years and 45%–46% against hospitalizations among immunocompetent adults aged ≥65 years, compared with not receiving a 2024–2025 vaccine dose. VE against hospitalizations in immunocompromised adults aged ≥65 years was 40%.

**What are the implications for public health practice?**

These findings indicate that 2024–2025 COVID-19 vaccination provides additional protection against COVID-19–associated ED/UC encounters and hospitalization, versus no 2024–2025 vaccination and support CDC and ACIP recommendations that all persons aged ≥6 months receive 2024–2025 COVID-19 vaccination.

## Abstract

COVID-19 vaccination averted approximately 68,000 hospitalizations during the 2023–24 respiratory season. In June 2024, CDC and the Advisory Committee on Immunization Practices (ACIP) recommended that all persons aged ≥6 months receive a 2024–2025 COVID-19 vaccine, which targets Omicron JN.1 and JN.1-derived sublineages. Interim effectiveness of 2024–2025 COVID-19 vaccines was estimated against COVID-19–associated emergency department (ED) or urgent care (UC) visits during September 2024–January 2025 among adults aged ≥18 years in one CDC-funded vaccine effectiveness (VE) network, against COVID-19–associated hospitalization in immunocompetent adults aged ≥65 years in two networks, and against COVID-19–associated hospitalization among adults aged ≥65 years with immunocompromising conditions in one network. Among adults aged ≥18 years, VE against COVID-19–associated ED/UC visits was 33% (95% CI = 28%–38%) during the first 7–119 days after vaccination. Among immunocompetent adults aged ≥65 years from two CDC networks, VE estimates against COVID-19–associated hospitalization were 45% (95% CI = 36%–53%) and 46% (95% CI = 26%–60%) during the first 7–119 days after vaccination. Among adults aged ≥65 years with immunocompromising conditions in one network, VE was 40% (95% CI = 21%–54%) during the first 7–119 days after vaccination. These findings demonstrate that vaccination with a 2024–2025 COVID-19 vaccine dose provides additional protection against COVID-19–associated ED/UC encounters and hospitalizations compared with not receiving a 2024–2025 dose and support current CDC and ACIP recommendations that all persons aged ≥6 months receive a 2024–2025 COVID-19 vaccine dose.

## Introduction

During September 24, 2023–August 11, 2024, approximately 800,000 COVID-19–associated hospitalizations occurred in the United States (*1*); adults aged ≥65 years accounted for 70% of these hospitalizations (*2*). During 2024, the SARS-CoV-2 Omicron JN.1 and JN.1-derived lineages predominated and were genomically divergent from the XBB lineages on which the 2023–2024 COVID-19 vaccines were based. On June 27, 2024, CDC’s Advisory Committee on Immunization Practices (ACIP) recommended 2024–2025 COVID-19 vaccination with a Food and Drug Administration (FDA)–authorized or approved vaccine for all persons aged ≥6 months (*3*). In August 2024, FDA approved monovalent 2024–2025 COVID-19 vaccines by Moderna* and Pfizer-BioNTech^†^ (based on the SARS-CoV-2 Omicron KP.2 lineage) and authorized a monovalent 2024–2025 COVID-19 vaccine by Novavax^§^ (based on the SARS-CoV-2 Omicron JN.1 lineage), for persons aged ≥12 years. For a majority of adults, 1 2024–2025 vaccine dose is recommended, although persons with moderate or severe immunocompromise and adults aged ≥65 years are recommended to receive additional doses, depending on their vaccination history and time since receipt of their most recent dose.^¶^

This analysis estimated 2024–2025 COVID-19 vaccine effectiveness (VE) against COVID-19–associated emergency department (ED) or urgent care (UC) visits in one CDC-funded VE network and VE against COVID-19–associated hospitalization in two CDC-funded VE networks during September 2024–January 2025** among adults aged ≥18 years.

## Methods

### Data Source

Methods for VE analyses in the Virtual SARS-CoV-2, Influenza, and Other respiratory viruses Network (VISION) and Investigating Respiratory Viruses in the Acutely Ill (IVY) network have been reported (*4*,*5*). VISION is a multisite, electronic health care records (EHR)–based network including 373 ED/UCs and 241 hospitals in eight states.^††^ Eligible patients are those who have received molecular (e.g., real-time reverse transcription–polymerase chain reaction [RT-PCR]) or antigen testing for SARS-CoV-2 during the 10 days preceding or ≤72 hours after an eligible ED/UC encounter or hospital admission.^§§^ COVID-19 vaccination history is ascertained from state or jurisdictional registries, EHRs and, in a subset of sites, medical claims data.^¶¶^

IVY is a multicenter, inpatient network of 26 hospitals in 20 U.S. states*** and prospectively enrolls adults aged ≥18 years with COVID-19–like illness^†††^ who receive molecular or antigen testing for SARS-CoV-2 within 10 days of illness onset and within 3 days of hospital admission. Nasal swabs are collected at enrollment for central RT-PCR testing for SARS-CoV-2 at Vanderbilt University Medical Center (Nashville, Tennessee); SARS-CoV-2–positive specimens are sent to the University of Michigan (Ann Arbor, Michigan) for whole genome sequencing to identify SARS-CoV-2 lineages. Demographic and clinical data are collected through EHR review and patient or proxy interview. COVID-19 vaccination history is ascertained from state or jurisdictional registries, EHRs, and plausible self-report based on known location and dates of vaccination.

In both analyses, persons who had received the 2024–2025 COVID-19 vaccine ≥7 days before the encounter index date (VISION) or illness onset date (IVY) were considered vaccinated. Those who had not received the 2024–2025 COVID-19 vaccine (regardless of previous COVID-19 vaccination or infection history) were considered not vaccinated and served as comparators.

### Data Analysis

The VISION and IVY networks conducted separate VE analyses using test-negative designs (*4*,*5*). In both analyses, adults aged ≥18 years with COVID-19–like illness who 1) had a medical encounter at an ED/UC (VISION only) or 2) were hospitalized (VISION and IVY) at a participating facility were included. Case-patients were those who received a positive SARS-CoV-2 molecular or antigen test result, and control patients were those who received a negative SARS-CoV-2 molecular test result. Participants were excluded if they 1) had received a 2024–2025 COVID-19 vaccine <7 days or ≥120 days before their eligible ED/UC encounter or hospitalization, 2) had received a 2024–2025 COVID-19 vaccine dose <2 months after receiving a previous COVID-19 vaccine dose, or 3) were immunocompetent persons who had received more than 1 2024–2025 COVID-19 vaccine dose. COVID-19 case-patients were also excluded if they were co-infected with influenza or respiratory syncytial virus (RSV) at the time of their COVID-19–like illness encounter. Because of potential confounding from correlated vaccination behaviors, control patients with a positive or indeterminant influenza test result (adults ≥18 years) or a positive RSV test result (adults ≥60 years) were excluded from the primary analysis (*6*,*7*). Previous SARS-CoV-2 infections are incompletely documented in medical records; therefore, patients were included regardless of prior SARS-CoV-2 infections.

Odds ratios (OR) and 95% CIs were estimated using multivariable logistic regression, comparing persons who received a 2024–2025 COVID-19 vaccine dose with those who did not among case- and control patients, regardless of previous COVID-19 vaccination. VE models were adjusted a priori for age, sex, race and ethnicity, calendar time, and geographic region.^§§§^ VE was calculated as (1 − adjusted OR) x 100% during the first 7–119 days since receipt of a 2024–2025 COVID-19 vaccine dose and separately during the first 7–59 days and 60–119 days since receipt of a dose. For ED/UC encounters, VE was estimated for persons aged ≥18 years, 18–64 years, and ≥65 years (Supplementary Table 1, https://stacks.cdc.gov/view/cdc/176586). Statistical power to estimate VE against hospitalization was limited in adults aged 18–64 years; therefore, VE against hospitalization was only estimated for adults aged ≥65 years in both networks. In the IVY network, VE against hospitalization was estimated in immunocompetent adults due to limited statistical power to assess VE for immunocompromised adults; in VISION, VE was estimated for all adults in the ED/UC setting and separately for adults with and without immunocompromising conditions in the hospital setting.^¶¶¶^ The distribution of case- and control patients aged 5–17 years was explored in VISION; however, statistical power was limited in both the ED/UC and hospital settings, so frequencies are described without VE estimation (Supplementary Table 2; https://stacks.cdc.gov/view/cdc/176592).

Analyses were conducted using R software (version 4.3.2; R Foundation) for the VISION analysis and R software (version 4.4.0; R Foundation) for the IVY network analysis. This activity was reviewed by CDC, deemed not research, and was conducted consistent with applicable federal law and CDC policy.****

## Results

### 2024–2025 COVID-19 VE Against COVID-19–associated ED/UC Visits, VISION

Among adults aged ≥18 years in VISION, 137,543 ED/UC encounters met criteria for inclusion in the analyses, including 10,459 (8%) case-patients and 127,084 (92%) control patients (Table 1). Effectiveness of a 2024–2025 COVID-19 vaccination against a COVID-19–associated ED/UC visit was 33% (95% CI = 28%–38%) during the first 7–119 days after vaccination, 36% (95% CI = 29%–42%) during the first 7–59 days after vaccination, and 30% (95% CI = 22%–37%) during the 60–119 days after vaccination (Table 2).

**TABLE 1:** Characteristics of emergency department and urgent care encounters and hospitalizations among adults aged ≥18 years with COVID-19–like illness, by COVID-19 case status and CDC vaccine effectiveness network — VISION and IVY Networks,* September 2024–January 2025

Characteristic	VE network and setting, no. (column %)								
	VISION ED/UC encounters, all adults aged ≥18 years			VISION hospitalizations, all adults aged ≥65 years			IVY hospitalizations, immunocompetent adults aged ≥65 years		
	Total	COVID-19 case-patients	COVID-19 control patients	Total	COVID-19 case-patients	COVID-19 control patients	Total	COVID-19 case-patients	COVID-19 control patients
**All encounters**	**137,543**	**10,459**	**127,084**	**34,411**	**2,846**	**31,565**	**1,929**	**683**	**1,246**
**2024–2025 COVID-19 vaccination status**									
No 2024–2025 dose^†^	**118,517 (86)**	9,545 (91)	108,972 (86)	**27,623 (80)**	2,540 (89)	25,083 (79)	**1,635 (85)**	614 (90)	1,021 (82)
**Received 2024–2025 dose**									
7–119 days earlier	**19,026 (14)**	914 (9)	18,112 (14)	**6,788 (20)**	306 (11)	6,482 (21)	**294 (15)**	69 (10)	225 (18)
7–59 days earlier	**10,269 (7)**	480 (5)	9,789 (8)	**3,904 (11)**	179 (6)	3,725 (12)	**146 (8)**	41 (6)	105 (8)
60–119 days earlier	**8,757 (6)**	434 (4)	8,323 (7)	**2,884 (8)**	127 (4)	2,757 (9)	**148 (8)**	28 (4)	120 (10)
**Median age, yrs (IQR)**	**53 (34–72)**	58 (37–74)	53 (34–71)	**78 (72–84)**	79 (73–86)	78 (71–84)	**77 (71–84)**	78 (72, 85)	76 (70, 83)
**Age group, yrs^§^**									
18–64	**88,858 (65)**	6,113 (58)	82,745 (65)	**—**	**—**	**—**	**—**	**—**	**—**
≥65	**48,685 (35)**	4,346 (42)	44,339 (35)	**34,411 (100)**	2,846 (100)	31,565 (100)	**1,929 (100)**	683 (100)	1,246 (100)
**Female sex**	**83,641 (61)**	6,275 (60)	77,366 (61)	**18,274 (53)**	1,412 (50)	16,862 (53)	** 1,050 (54)**	374 (55)	676 (54)
**Race and ethnicity**									
Black or African American, NH	**15,003 (11)**	794 (8)	14,209 (11)	**2,575 (7)**	156 (5)	2,419 (8)	** 370 (19)**	120 (18)	250 (20)
White, NH	**83,282 (61)**	7,256 (69)	76,026 (60)	**25,811 (75)**	2,281 (80)	23,530 (75)	** 1,223 (63)**	447 (65)	776 (62)
Hispanic or Latino, any race	**20,461 (15)**	1,255 (12)	19,206 (15)	**2,640 (8)**	183 (6)	2,457 (8)	**184 (10)**	59 (9)	125 (10)
Other, NH^¶^	**14,014 (10)**	897 (9)	13,117 (10)	**2,858 (8)**	188 (7)	2,670 (8)	**89 (5)**	34 (5)	55 (4)
Unknown**	**4,783 (3)**	257 (2)	4,526 (4)	**527 (2)**	38 (1)	489 (2)	**63 (3)**	23 (3)	40 (3)
**HHS region^††^**									
1	**0**	0	0	**0**	0	0	** 614 (32)**	235 (34)	379 (30)
2	**0**	0	0	**0**	0	0	**104 (5)**	23 (3)	81 (7)
3	**0**	0	0	**0**	0	0	**21 (1)**	10 (2)	11 (1)
4	**0**	0	0	**0**	0	0	**279 (15)**	93 (14)	186 (15)
5	**45,211 (33)**	3,416 (33)	41,795 (33)	**13,844 (40)**	1,261 (44)	12,583 (40)	**248 (13)**	115 (17)	133 (11)
6	**0**	0	0	**0**	0	0	**144 (8)**	33 (5)	111 (9)
7	**0**	0	0	**0**	0	0	**52 (3)**	11 (2)	41 (3)
8	**33,345 (24)**	4,519 (43)	28,826 (23)	**6,217 (18)**	696 (24)	5,521 (17)	**254 (13)**	86 (13)	168 (14)
9	**48,738 (35)**	1,728 (17)	47,010 (37)	**12,574 (37)**	766 (27)	11,808 (37)	**154 (8)**	54 (8)	100 (8)
10	**10,249 (7)**	796 (8)	9,453 (7)	**1,776 (5)**	123 (4)	1,653 (5)	**59 (3)**	23 (3)	36 (3)
**No. of organ systems with a chronic medical condition, median (IQR)^§§^**	**0 (0–1)**	0 (0–1)	0 (0–1)	**3 (2–4)**	3 (2–4)	3 (2–4)	**3 (2, 4)**	2 (2, 3)	3 (2, 4)
**Immunocompromised^¶¶^**	**—**	—	—	**8,192 (24)**	598 (21)	7,594 (24)	—	—	—
**Month/Yr. of COVID-19–associated ED/UC encounter or hospitalization**									
Sep 2024	**28,086 (20)**	3,675 (35)	24,411 (19)	**7,723 (22)**	982 (35)	6,741 (21)	** 508 (26)**	229 (34)	279 (22)
Oct 2024	**28,364 (21)**	1,927 (18)	26,437 (21)	**7,641 (22)**	557 (20)	7,084 (22)	**377 (20)**	147 (22)	230 (19)
Nov 2024	**28,040 (20)**	1,465 (14)	26,575 (21)	**7,751 (23)**	408 (14)	7,343 (23)	**312 (16)**	114 (17)	198 (16)
Dec 2024	**38,148 (28)**	2,712 (26)	35,436 (28)	**9,063 (26)**	771 (27)	8,292 (26)	**394 (20)**	125 (18)	269 (22)
Jan 2025	**14,905 (11)**	680 (7)	14,225 (11)	**2,233 (6)**	128 (4)	2,105 (7)	**338 (18)**	68 (10)	270 (22)

**Abbreviations:** ED = emergency department; EHR = electronic health care records; HHS = U.S. Department of Health and Human Services; IVY = Investigating Respiratory Viruses in the Acutely Ill; NH = non-Hispanic; UC = urgent care; VE = vaccine effectiveness; VISION = Virtual SARS-CoV-2, Influenza, and Other respiratory viruses Network.

* Sites from the CDC-funded VISION that contributed data for this analysis were HealthPartners (Minnesota and Wisconsin), Intermountain Health (Utah), Kaiser Permanente Northern California (California), Kaiser Permanente Northwest (Oregon and Washington), Regenstrief Institute (Indiana), and University of Colorado (Colorado). Sites from the CDC-funded IVY network that contributed data for this analysis were Barnes-Jewish Hospital (St. Louis, Missouri), Baylor Scott & White Medical Center (Temple, Texas), Baylor University Medical Center (Dallas, Texas), Baystate Medical Center (Springfield, Massachusetts), Beth Israel Deaconess Medical Center (Boston, Massachusetts), Cleveland Clinic (Cleveland, Ohio), Emory University Medical Center (Atlanta, Georgia), Hennepin County Medical Center (Minneapolis, Minnesota), Henry Ford Health (Detroit, Michigan), Intermountain Medical Center (Murray, Utah), Johns Hopkins Hospital (Baltimore, Maryland), Montefiore Medical Center (New York, New York), Oregon Health and Science University Hospital (Portland, Oregon), Ronald Reagan UCLA Medical Center (Los Angeles, California), Stanford University Medical Center (Stanford, California), The Ohio State University Wexner Medical Center (Columbus, Ohio), UCHealth University of Colorado Hospital (Aurora, Colorado), University of Arizona Medical Center (Tucson, Arizona), University of Iowa Hospitals (Iowa City, Iowa), University of Miami Medical Center (Miami, Florida), University of Michigan Hospital (Ann Arbor, Michigan), University of Utah (Salt Lake City, Utah), University of Washington (Seattle, Washington), Vanderbilt University Medical Center (Nashville, Tennessee), Wake Forest University Baptist Medical Center (Winston-Salem, North Carolina), and Yale University (New Haven, Connecticut).

^†^ The “no 2024–2025 dose” group included all eligible persons who did not receive 2024–2025 COVID-19 vaccine dose, regardless of number of previous doses (if any) received.

^§^ In VISION, a total of 18,289 eligible hospitalizations were reported in adults aged 18–64 years, including 804 (4%) case-patients and 17,485 (96%) control patients. Of the hospitalized case-patients, 35 (4%) had received a 2024–2025 COVID-19 vaccine. Of the hospitalized control patients, 1,277 (7%) had received a 2024–2025 COVID-19 vaccine. In IVY, a total of 1,446 eligible hospitalizations were reported in adults aged 18–64 years, including 342 (24%) case-patients and 1,104 (76%) control patients. Of the case-patients aged 18–64 years, 16 (5%) had received a 2024–2025 COVID-19 vaccine. Of the control patients aged 18–64 years, 69 (6%) had received a 2024–2025 COVID-19 vaccine.

^¶^ For VISION, “Other, non-Hispanic” race includes persons reporting non-Hispanic ethnicity and any of the following for race: American Indian or Alaska Native, Asian, Native Hawaiian or Pacific Islander, Middle Eastern or North African, other races not listed, and multiple races. Because of small numbers, these categories were combined. For IVY, “Other race, non-Hispanic” includes Asian, American Indian or Alaska Native, Native Hawaiian or other Pacific Islander and patients who self-reported their race and ethnicity as, “Other”; these groups were combined because of small counts.

** For VISION, “Unknown” includes persons with missing race and ethnicity in their EHR. For IVY, “Unknown” refers to patients who did not report their race and ethnicity.

^††^ In VISION, geographic region was included in the model based on site-defined geographic cluster of the final discharge facility of the encounter. In IVY, geographic region was included in the model based on HHS region. HHS regions are included to illustrate geographic spread across both networks. Regions are defined by HHS. States included in each region are available at https://www.hhs.gov/about/agencies/iea/regional-offices/index.html. VISION sites included were located as follows: *Region 5:* HealthPartners (Minnesota and Wisconsin) and Regenstrief Institute (Indiana); *Region 8*: Intermountain Healthcare (Utah) and University of Colorado (Colorado); *Region 9*: Kaiser Permanente Northern California (California); and *Region 10*: Kaiser Permanente Northwest (Oregon and Washington). IVY network sites were located as follows: *Region 1*: Baystate Medical Center (Springfield, Massachusetts), Beth Israel Deaconess Medical Center (Boston, Massachusetts), and Yale University (New Haven, Connecticut); *Region 2*: Montefiore Medical Center (New York, New York); *Region 3*: Johns Hopkins Hospital (Baltimore, Maryland); *Region 4*: Emory University Medical Center (Atlanta, Georgia), University of Miami Medical Center (Miami, Florida), Vanderbilt University Medical Center (Nashville, Tennessee), and Wake Forest University Baptist Medical Center (Winston-Salem, North Carolina); *Region 5*: Cleveland Clinic (Cleveland, Ohio), Hennepin County Medical Center (Minneapolis, Minnesota), Henry Ford Health (Detroit, Michigan), The Ohio State University Wexner Medical Center (Columbus, Ohio), and University of Michigan Hospital (Ann Arbor, Michigan); *Region 6*: Baylor Scott & White Medical Center (Temple, Texas) and Baylor University Medical Center (Dallas, Texas); *Region 7*: Barnes-Jewish Hospital (St. Louis, Missouri) and University of Iowa Hospitals (Iowa City, Iowa); *Region 8*: Intermountain Medical Center (Murray, Utah), UCHealth University of Colorado Hospital (Aurora, Colorado), and University of Utah (Salt Lake City, Utah); *Region 9*: Stanford University Medical Center (Stanford, California), Ronald Reagan UCLA Medical Center (Los Angeles, California), and University of Arizona Medical Center (Tucson, Arizona); and *Region 10*: Oregon Health and Science University Hospital (Portland, Oregon) and University of Washington (Seattle, Washington).

^§§^ VISION underlying medical condition categories included pulmonary, cardiovascular, cerebrovascular, neurologic or musculoskeletal, hematologic, endocrine, renal, and gastrointestinal. IVY network underlying medical condition categories included pulmonary, cardiovascular, neurologic, hematologic, endocrine, kidney, gastrointestinal, and autoimmune.

^¶¶^ Immunocompromised status is not evaluated for ED/UC encounters because of a higher likelihood of incomplete discharge diagnosis codes in this setting. In IVY, a total of 656 eligible hospitalizations were reported in adults aged ≥65 years with immunocompromise, including 178 (27%) case-patients and 478 (73%) control patients. Of the case-patients aged ≥65 years with immunocompromise, 24 (13%) had received a 2024–2025 COVID-19 vaccine. Of the control patients aged ≥65 years with immunocompromise, 102 (21%) had received a 2024–2025 COVID-19 vaccine. Immunocompromised adults were excluded from the IVY Network’s VE analyses due to limited sample size.

**TABLE 2:** Effectiveness of 2024–2025 COVID-19 vaccination against COVID-19–associated emergency department or urgent care encounters, by age group — VISION, September 2024–January 2025

Age group/COVID-19 vaccination dosage pattern	COVID-19 case-patients No. (col %)	COVID-19 control patients No. (col %)	Median interval since last dose for vaccinated, days (IQR)	VE %* (95% CI)
**≥18 yrs**				
No 2024–2025 dose^†^ (Ref)	9,545 (91)	108,972 (86)	998 (539–1,142)	Ref
Received 2024–2025 dose				
7–119 days earlier	914 (9)	18,112 (14)	55 (32–80)	33 (28–38)
7–59 days earlier	480 (5)	9,789 (8)	33 (20–46)	36 (29–42)
60–119 days earlier	434 (4)	8,323 (7)	82 (71–97)	30 (22–37)
**18–64 yrs**				
No 2024–2025 dose^†^ (Ref)	5,860 (96)	76,792 (93)	1,042 (751–1,180)	Ref
Received 2024–2025 dose				
7–119 days earlier	253 (4)	5,953 (7)	53 (29–77)	30 (20–39)
7–59 days earlier	134 (2)	3,379 (4)	32 (20–45)	36 (23–46)
60–119 days earlier	119 (2)	2,574 (3)	81 (70–95)	21 (5–35)
**≥65 yrs**				
No 2024–2025 dose^†^ (Ref)	3,685 (85)	32,180 (73)	750 (346–1,076)	Ref
Received 2024–2025 dose				
7–119 days earlier	661 (15)	12,159 (27)	57 (33–82)	35 (29–41)
7–59 days earlier	346 (8)	6,410 (14)	34 (21–47)	36 (28–44)
60–119 days earlier	315 (7)	5,749 (13)	83 (71–97)	34 (25–42)

**Abbreviations:** Ref = referent group; VE = vaccine effectiveness; VISION = Virtual SARS-CoV-2, Influenza, and Other respiratory viruses Network.

* VE was calculated by comparing the odds of 2024–2025 COVID-19 vaccination among case-patients and control patients using the following equation: (1 – adjusted odds ratio) x 100%. Odds ratios were estimated by multivariable logistic regression. The odds ratio was adjusted for age, sex, race and ethnicity, calendar day, and geographic region. ^† ^The “no 2024–2025 dose” group included all eligible persons who did not receive a 2024–2025 COVID-19 vaccine dose, regardless of number of previous COVID-19 vaccine doses (if any) received.

### 2024–2025 COVID-19 VE Against COVID-19–associated Hospitalization, VISION and IVY Networks Among Older Adults

Among adults aged ≥65 years without immunocompromising conditions in VISION, 26,219 hospitalizations met criteria for inclusion in analyses, including 2,248 (9%) case-patients and 23,971 (91%) control patients. VE of a 2024–2025 COVID-19 vaccine dose against COVID-19–associated hospitalization was 45% (95% CI = 36%–53%) a median interval of 53 days since receipt of a 2024–2025 COVID-19 vaccine dose (Table 3). Among adults aged ≥65 years with immunocompromising conditions in VISION, 8,192 hospitalizations met criteria for inclusion in analyses, including 598 (7%) case-patients and 7,594 (93%) control patients. VE was 40% (95% CI = 21%–54%), a median interval of 53 days after receipt of a 2024–2025 COVID-19 vaccination. Among adults aged ≥65 years without immunocompromising conditions in the IVY network, 1,929 met inclusion criteria, including 683 (35%) case-patients and 1,246 (65%) control patients. VE against COVID-19–associated hospitalization was 46% (95% CI = 26%–60%), a median of 60 days after receipt of a 2024–2025 COVID-19 vaccine dose.

**TABLE 3:** Effectiveness of 2024–2025 COVID-19 vaccination against COVID-19–associated hospitalization among adults aged ≥65 years — VISION and IVY Networks, September 2024–January 2025

VE network/Immunocompromise status/ COVID-19 vaccination dosage pattern	COVID-19 case-patients No. (col %)	COVID-19 control patients No. (col %)	Median interval since last dose for vaccinated, days (IQR)	VE %* (95% CI)
**VISION, immunocompetent**				
**No 2024–2025 dose^†^ (Ref)**	2,016 (90)	19,198 (80)	775 (357–1,084)	**Ref**
**Received 2024–2025 dose**				
7–119 days earlier	232 (10)	4,773 (20)	53 (30–77)	**45 (36–53)**
7–59 days earlier	129 (6)	2,759 (12)	33 (20–46)	**42 (30–52)**
60–119 days earlier	103 (5)	2,014 (8)	81 (70–94)	**48 (36–58)**
**VISION, immunocompromised**				
**No 2024–2025 dose^†^ (Ref)**	524 (88)	5,885 (78)	720 (343–1,064)	**Ref**
**Received 2024–2025 dose**				
7–119 days earlier	74 (12)	1,709 (22)	53 (31–78)	**40 (21–54)**
**IVY network, immunocompetent**				
**No 2024–2025 dose^†^ (Ref)**	614 (90)	1,021 (82)	^—§^	**Ref**
**Received 2024–2025 dose**				
7–119 days earlier	69 (10)	225 (18)	60 (31–85)	**46 (26–60)**
7–59 days earlier	41 (6)	105 (9)	31 (20–45)	**42 (14–61)**
60–119 days earlier	28 (4)	120 (11)	85 (72–98)	**47 (17–67)**

**Abbreviations:** Ref = referent group; VE = vaccine effectiveness; VISION = Virtual SARS-CoV-2, Influenza, and Other respiratory viruses Network; IVY = Investigating Respiratory Viruses in the Acutely Ill.

* VE was calculated by comparing the odds of 2024–2025 COVID-19 vaccination in case-patients and control patients using the equation: (1 − adjusted odds ratio) x 100%. Odds ratios were estimated by multivariable logistic regression. For VISION, the odds ratio was adjusted for age, sex, race and ethnicity, calendar day, and geographic region. For IVY, the odds ratio was adjusted for age, sex, race and ethnicity, geographic region (U.S. Department of Health and Human Services region), and calendar time (biweekly intervals).

^†^ The “no 2024–2025 dose” group included all eligible persons who did not receive a 2024–2025 COVID-19 vaccine dose, regardless of number of previous COVID-19 vaccine doses (if any) received.

^§^ Median interval from last dose for persons who received previous doses of COVID-19 vaccine but did not receive a 2024–2025 COVID-19 vaccine dose was not available in the IVY network.

### Whole Genome Sequencing of SARS-CoV-2 Specimens, IVY Network

Among adults aged ≥18 years in the IVY network, 653 SARS-CoV-2–positive specimens collected during September 1, 2024–December 31, 2024 were successfully sequenced; 55 (8.4%) had JN.1-like spike proteins, 92 (14.1%) had KP.2-like proteins, 340 (52.1%) had KP.3-like proteins, 126 (19.3%) had XEC-like proteins, and 40 (6.1%) had other spike proteins.^††††^ Similarly, among 6,491 SARS-CoV-2–positive specimens collected during the same period and sequenced by CDC as part of national genomic surveillance,^§§§§^ 928 (14.3%) had JN.1-like spike proteins, 982 (15.1%) had KP.2-like proteins, 3,430 (52.8%) had KP.3-like proteins, 894 (13.8%) XEC-like proteins, and 257 (4.0%) had other spike proteins.

## Discussion

During September 2024–January 2025, 2024–2025 COVID-19 vaccination provided additional protection against COVID-19–associated ED/UC encounters and hospitalizations among adults with and without immunocompromising conditions, compared with not receiving a 2024–2025 COVID-19 vaccine dose. These results support current CDC recommendations for 2024–2025 COVID-19 vaccination, irrespective of previous COVID-19 vaccination and infection history, and represent the added benefit of 2024–2025 COVID-19 vaccination above existing protection from previous vaccination or infection (*3*).

During the analytic period, the primary circulating SARS-CoV-2 lineages were descendants of the Omicron JN.1 lineage, including KP.2, KP.3, and XEC.^¶¶¶¶^ XEC is closely related to the KP.2 and JN.1 strains in the 2024–2025 COVID-19 vaccines, which might account for the sustained protection from COVID-19 vaccination observed during the analysis period, despite the emergence and increasing prevalence of XEC. Starting in January 2025, prevalence of LP.8.1 (a JN.1 and KP.1.1 descendent) began to increase, accounting for 31% of sequences in CDC’s national genomic surveillance as of February 15, 2025. The pace and frequency with which new SARS-CoV-2 lineages have become predominant underscores the need for ongoing monitoring of COVID-19 VE and genomic surveillance.

COVID-19–associated hospitalization rates during the time frame of this analysis were relatively low compared with those during previous years, precluding estimation of VE against critical illness (i.e., intensive care unit admission, invasive mechanical ventilation, or death); VE against these outcomes has historically been higher and more sustained than that against less severe outcomes (*4*,*5*,*8*). Because of both lower hospitalization rates and lower vaccination rates,***** VE could not be estimated for children and adolescents aged 5–17 years for either outcome or for adults aged 18–64 years against hospitalization. Analyses from previous years have indicated that COVID-19 vaccines provide similar protection across age groups. For the 2023–2024 COVID-19 vaccines, VE against ED/UC encounters during the first 60–179 days after vaccination was 24% (95% CI = -31% to 56%) for children aged 9 months–4 years, 50% (95% CI = 22%–68%) for children and adolescents aged 5 years–17 years, 24% (95% CI = 17%–31%) for adults aged 18–64 years, and 25% (95% CI = 20%–30%) for adults aged ≥65 years.^†††††^

Previous SARS-CoV-2 infection contributes protection against future disease, although protection wanes over time (*9*). An increase in SARS-CoV-2 circulation in the United States during late summer 2024, just before the 2024–2025 COVID-19 vaccines were approved and authorized, might have resulted in higher population-level immunity against JN.1-lineage strains, which could have resulted in lower measured VE than would have been detected in a population with less recent infection. Analyses did not account for previous SARS-CoV-2 infection or previous COVID-19 vaccination (e.g., original monovalent, bivalent, or 2023–2024 doses). VE should therefore be interpreted as the added benefit of 2024–2025 COVID-19 vaccination in a population with high levels of infection-induced immunity, vaccine-induced immunity, or both.

### Limitations

The findings in this report are subject to at least four limitations. First, although case-patients were those who met a COVID-19–like illness definition and had a positive SARS-CoV-2 test result, they might have visited ED/UCs or been hospitalized for reasons other than COVID-19, which might have lowered VE estimates. Second, misclassification of vaccination status was possible, which would likely result in underestimation of VE if the misclassification was nondifferential. Third, lack of statistical power prevented estimation of VE in some strata, including younger age groups. Finally, although analyses were adjusted for some relevant confounders, residual confounding from other factors, such as behavioral modifications to prevent SARS-CoV-2 exposure and outpatient antiviral treatment for COVID-19, might remain.

### Implications for Public Health Practice

In this analysis, receipt of a 2024–2025 COVID-19 vaccine dose provided additional protection against COVID-19–associated ED/UC visits and hospitalization among adults with and without immunocompromise. These results support CDC and ACIP recommendations for 2024–2025 COVID-19 vaccination (*3*). CDC continues to monitor VE of 2024–2025 COVID-19 vaccines.

## Footnotes

*
https://www.fda.gov/vaccines-blood-biologics/spikevax

†
https://www.fda.gov/vaccines-blood-biologics/comirnaty

§
https://www.fda.gov/vaccines-blood-biologics/coronavirus-covid-19-cber-regulated-biologics/novavax-covid-19-vaccine-adjuvanted

¶
https://www.cdc.gov/vaccines/covid-19/clinical-considerations/covid-19-vaccines-us.html

** The VISION analysis included ED/UC encounters and hospitalizations during September 1, 2024–January 21, 2025. The IVY network analysis included hospitalized patients admitted during September 1, 2024–January 30, 2025.

†† Sites from the CDC-funded VISION that contributed data for this analysis were HealthPartners (Minnesota and Wisconsin), Intermountain Health (Utah), Kaiser Permanente Northern California (California), Kaiser Permanente Northwest (Oregon and Washington), Regenstrief Institute (Indiana), and University of Colorado (Colorado).

§§ Eligible ED/UC encounters or hospital admissions were those for COVID-19–like illness, obtained using *International Classification of Diseases, Tenth Revision* (ICD-10) discharge codes. The specific codes used were COVID-19 pneumonia: J12.81 and J12.82; influenza pneumonia: J09.X1, J10.0*, J11.0*, and other viral pneumonia: J12*; bacterial and other pneumonia: J13, J14, J15*, J16*, J17, and J18*; influenza disease: J09*, J10.1, J10.2, J10.8*, J11.1, J11.2, and J11.8*; acute respiratory distress syndrome: J80; chronic obstructive pulmonary disease with acute exacerbation: J44.1; acute asthma exacerbation: J45.21, J45.22, J45.31, J45.32, J45.41, J45.42, J45.51, J45.52, J45.901, and J45.902; respiratory failure: J96.0*, J96.2*, R09.2, and J96.9*; other acute lower respiratory tract infections: B97.4, J20*, J21*, J22, J40, J44.0, J41*, J42, J43*, J47*, J85*, and J86*; acute and chronic sinusitis: J01* and J32*; acute upper respiratory tract infections: J00*, J02*, J03*, J04*, J05*, and J06*; acute respiratory illness signs and symptoms: R04.2, R05, R05.1, R05.2, R05.4, R05.8, R05.9, R06.00, R06.02, R06.03, R06.1, R06.2, R06.8, R06.81, R06.82, R06.89, R07.1, R09.0*, R09.1, R09.2, R09.3, and R09.8*; acute febrile illness signs and symptoms: R50*, R50.81, R50.9, and R68.83; acute nonrespiratory illness signs and symptoms: M79.10, M79.18, R10.0, R10.1*, R10.2, R10.3*, R10.81*, R10.84, R10.9, R11.0, R11.10, R11.11, R11.15, R11.2, R19.7, R21*, R40.0, R40.1, R41.82, R43*, R51.9, R53.1, R53.81, R53.83, R57.9, and R65*; febrile convulsions: R56.0; viral and respiratory diseases complicating pregnancy, childbirth, and puerperium: O98.5*, O98.8*, O98.9*, O99.5*. All ICD-10 codes with * include all child codes under the specific parent code.

¶¶ National pharmacy chains were required to establish bidirectional linkage with jurisdictional immunization information systems (IISs) to support vaccine distribution early in the COVID-19 pandemic; thus, doses administered at pharmacies should be reported to IISs.

*** Sites from the CDC-funded IVY network that contributed data for this analysis were Barnes-Jewish Hospital (St. Louis, Missouri), Baylor Scott & White Medical Center (Temple, Texas), Baylor University Medical Center (Dallas, Texas), Baystate Medical Center (Springfield, Massachusetts), Beth Israel Deaconess Medical Center (Boston, Massachusetts), Cleveland Clinic (Cleveland, Ohio), Emory University Medical Center (Atlanta, Georgia), Hennepin County Medical Center (Minneapolis, Minnesota), Henry Ford Health (Detroit, Michigan), Intermountain Medical Center (Murray, Utah), Johns Hopkins Hospital (Baltimore, Maryland), Montefiore Medical Center (New York, New York), Oregon Health and Science University Hospital (Portland, Oregon), Ronald Reagan UCLA Medical Center (Los Angeles, California), Stanford University Medical Center (Stanford, California), The Ohio State University Wexner Medical Center (Columbus, Ohio), UCHealth University of Colorado Hospital (Aurora, Colorado), University of Arizona Medical Center (Tucson, Arizona), University of Iowa Hospitals (Iowa City, Iowa), University of Miami Medical Center (Miami, Florida), University of Michigan Hospital (Ann Arbor, Michigan), University of Utah (Salt Lake City, Utah), University of Washington (Seattle, Washington), Vanderbilt University Medical Center (Nashville, Tennessee), Wake Forest University Baptist Medical Center (Winston-Salem, North Carolina), and Yale University (New Haven, Connecticut).

††† In the IVY network analysis, COVID-19–like illness was defined as one or more of the following signs and symptoms: fever, cough, dyspnea, new or worsening findings on chest imaging consistent with pneumonia, or hypoxemia defined as SpO2 <92% on room air or supplemental oxygen to maintain SpO2 ≥92%. For patients on chronic oxygen therapy, hypoxemia was defined as SpO2 below baseline or an escalation of supplemental oxygen to maintain a baseline SpO2.

§§§ VISION regression models were adjusted for age, sex, race and ethnicity, calendar day, and geographic region with age and calendar day included as natural cubic splines. Geographic region was included in the model based on site-defined geographic cluster of the final discharge facility of the encounter. IVY network regression models were adjusted for age, sex, race and ethnicity, calendar time in biweekly intervals, and U.S. Department of Health and Human Services region.

¶¶¶ Immunocompromising conditions were obtained from ICD-10 discharge codes. The specific codes used were hematologic malignancy: C81.*, C82.*, C83.*, C84.*, C85.*, C86.*, C88.*, C90.*, C91.*, C92.*, C93.*, C94.*, C95.*, C96.*, D46.*, D61.0*, D70.0, D61.2, D61.9, and D71.*; solid malignancy: C00.*, C01.*, C02.*, C03.*, C04.*, C05.*, C06.*, C07.*, C08.*, C09.*, C10.*, C11.*, C12.*, C13.*, C14.*, C15.*, C16.*, C17.*, C18.*, C19.*, C20.*, C21.*, C22.*, C23.*, C24.*, C25.*, C26.*, C30.*, C31.*, C32.*, C33, C34.*, C37, C38.*, C39.*, C40.*, C41.*, C43.*, C45.*, C46.*, C47.*, C48.*, C49.*, C50.*, C51.*, C52, C53.*, C54.*, C55, C56.*, C57.*, C58, C60.*, C61, C62.*, C63.*, C64.*, C65.*, C66.*, C67.*, C68.*, C69.*, C70.*, C71.*, C72.*, C73, C74.*, C75.*, C76.*, C77.*, C78.*, C79.*, C7A.*, C7B.*, C80.*, Z51.0, Z51.1*, and C4A.*; transplant: T86.0*, T86.1*, T86.2*, T86.3*, T86.4*, T86.5*, T86.81*, T86.85*, D47.Z1, Z48.2*, and Z94.*, and Z98.85; rheumatologic/inflammatory disorders: D86.*, E85.1, E85.2, E85.3, E85.4, E85.8*, E85.9, G35, J67.9, L40.54, L40.59, L93.0, L93.2, L94.*, M05.*, M06.*, M07.*, M08.*, M30.*, M31.3*, M31.5, M32.*, M33.*, M34.*, M35.3, M35.89, M35.9, M46.0*, M46.1, M46.8*, and M46.9*; other intrinsic immune condition or immunodeficiency: D27.9, D72.89, D80.*, D81.0, D81.1, D81.2, D81.4, D81.5, D81.6, D81.7, D81.8*, D81.9, D82.*, D83.*, D84.*, D89.0, D89.1, D89.3, D89.4*, D89.8*, D89.9, K70.3*, K70.4*, K72.*, K74.3, K74.4, K74.5, K74.6*, N04.*, and R18.0; HIV: B20.*, B21.*, B22.*, B23.*, B97.35, O98.7*, and Z21. All ICD-10 codes with * include all child codes under the specific parent code.

**** 45 C.F.R. part 46.102(l)(2), 21 C.F.R. part 56; 42 U.S.C. Sect. 241(d); 5 U.S.C. Sect. 552a; 44 U.S.C. Sect. 3501 et seq.

†††† SARS-CoV-2 lineages during the period of this analysis were classified according to their clade assignment as follows: sequences with clades 24A and 23I were grouped together as JN.1-like lineages; clades 24G and 24B were grouped together as KP.2-like lineages; clades 24C and 24E were grouped together as KP.3-like lineages; clade 24F represented XEC lineage; and “Other” represents non-JN.1-derived or recombinant viruses detected during September 1–December 31, 2024.

§§§§ CDC national SARS-CoV-2 genomic surveillance includes samples sequenced by CDC and national testing laboratories contracted by CDC.

¶¶¶¶
https://covid.cdc.gov/covid-data-tracker/#variant-proportions

*****
https://www.cdc.gov/respvaxview/about

†††††
https://www.cdc.gov/acip/downloads/slides-2024-06-26-28/03-COVID-Link-Gelles-508.pdf
